# Supplementary figures and images for: Multimodal image fusion for enhanced vehicle identification in intelligent transport
Source: PeerJ Comput Sci. 2025 Oct 30;11:e3270. doi: 10.7717/peerj-cs.3270 (PMC12594218; doi:10.7717/peerj-cs.3270)

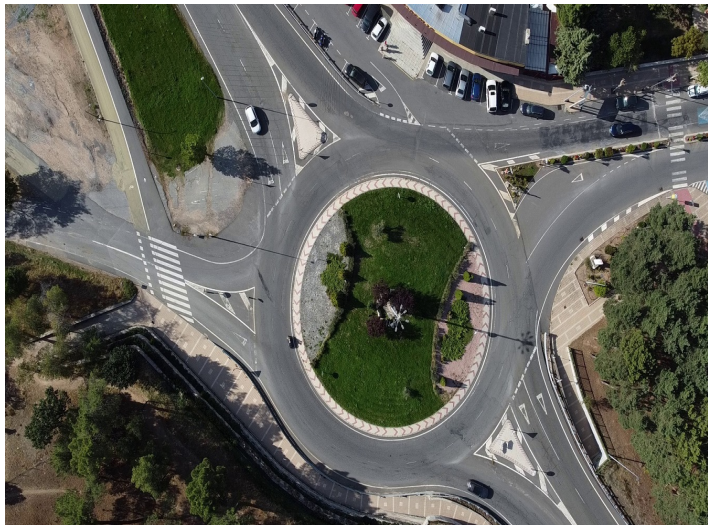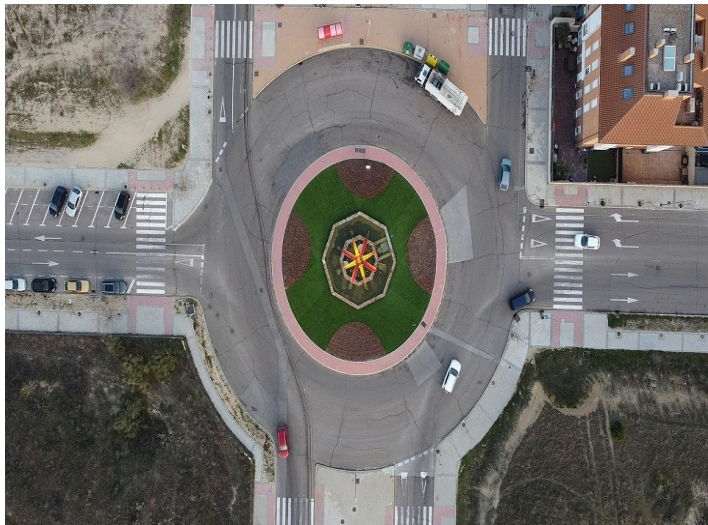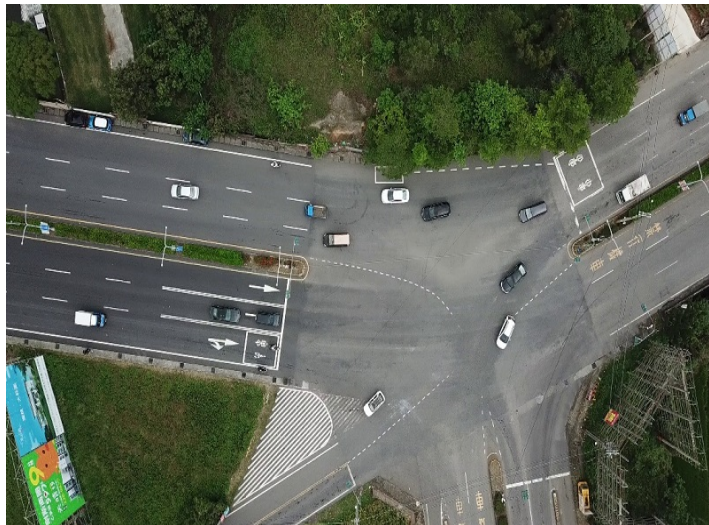

(a)

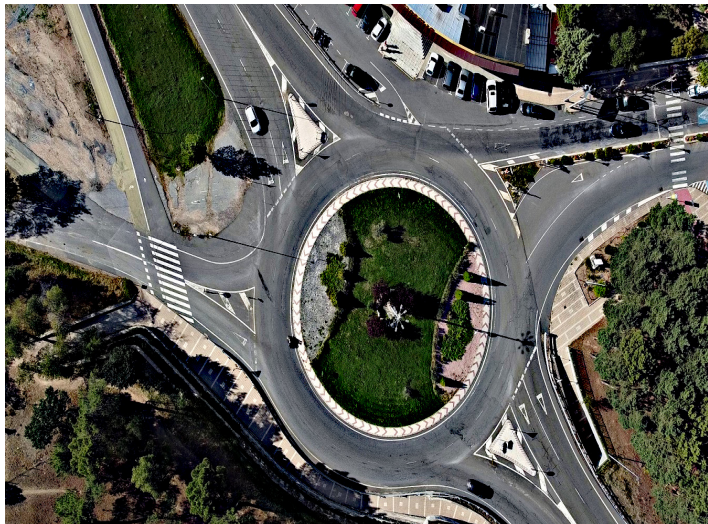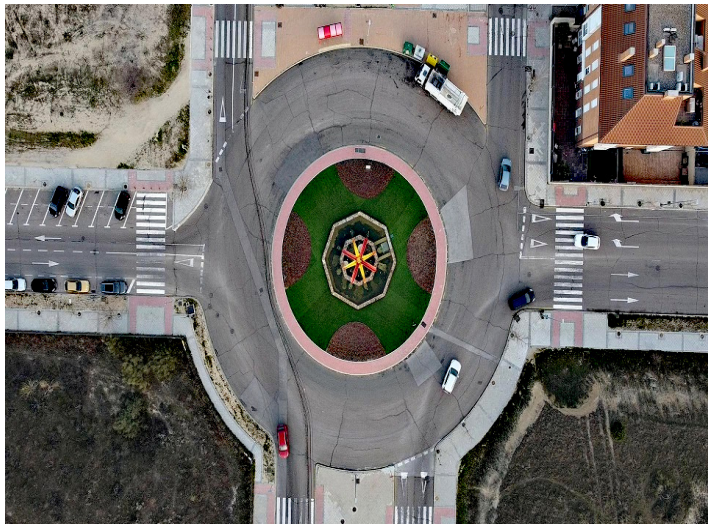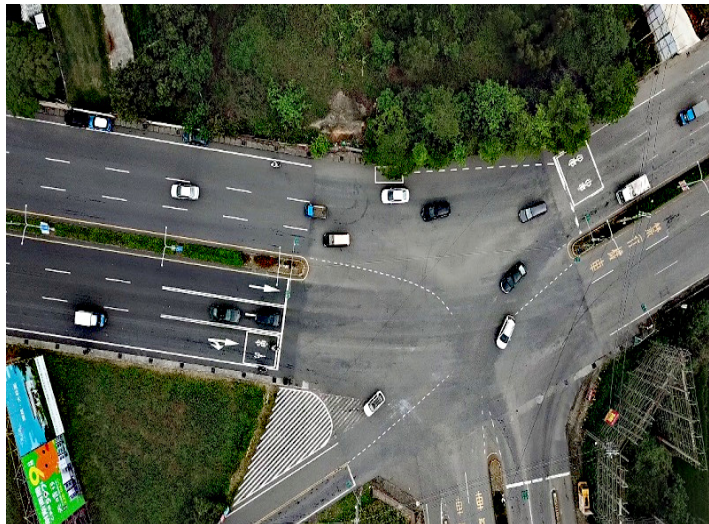

(a)

Supplement: Supplemental Information 1 [file peerj-cs-11-3270-s001.pdf]

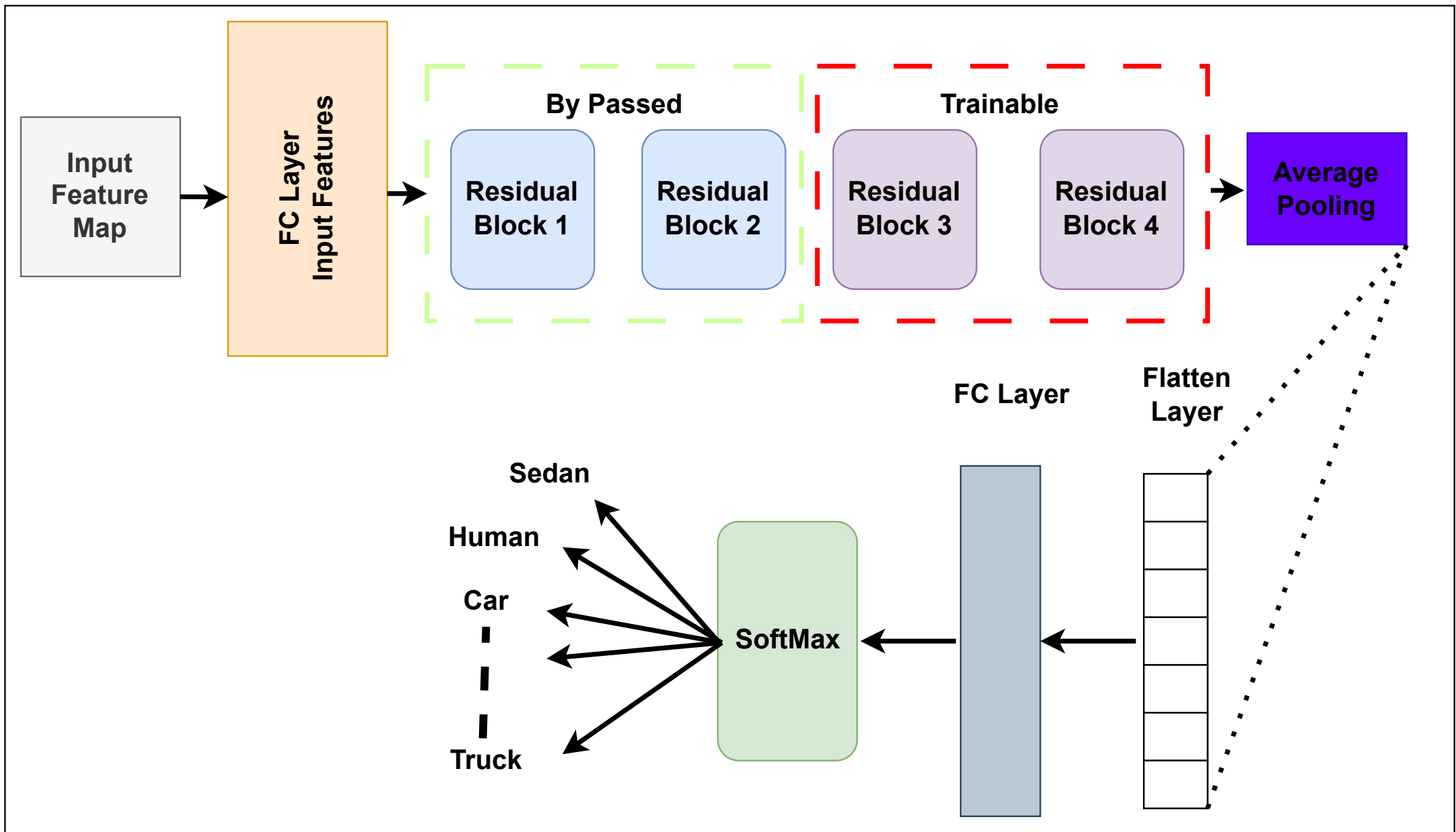

Supplement: Supplemental Information 2 [file peerj-cs-11-3270-s002.pdf]

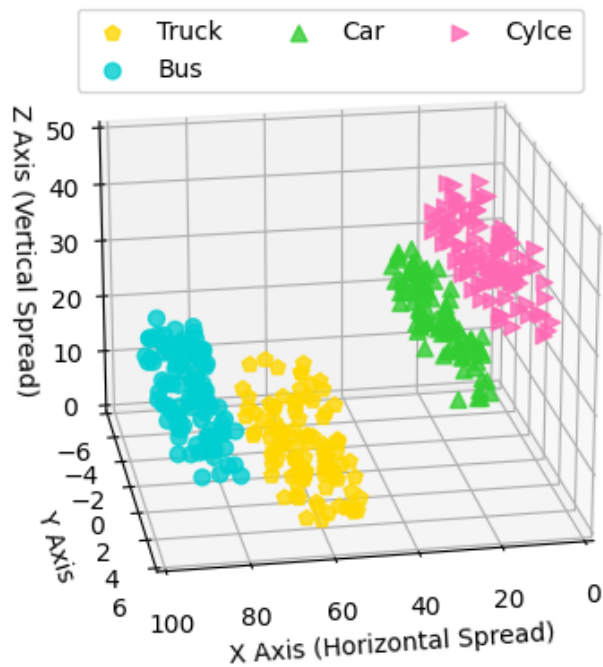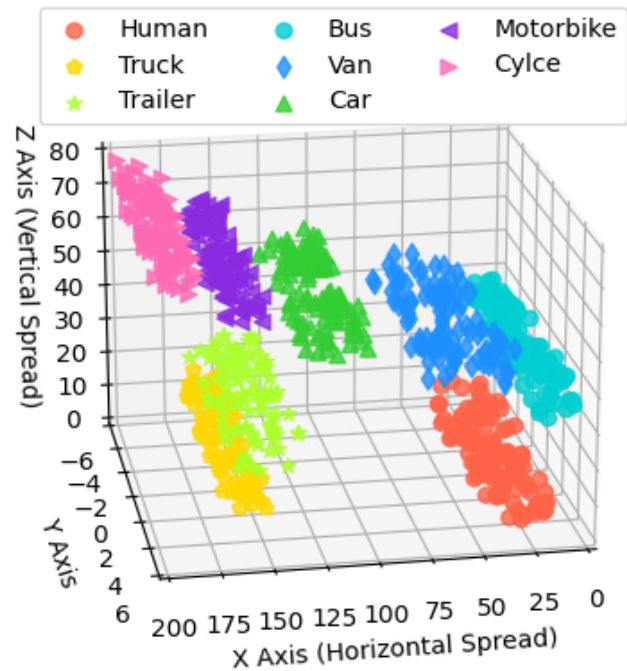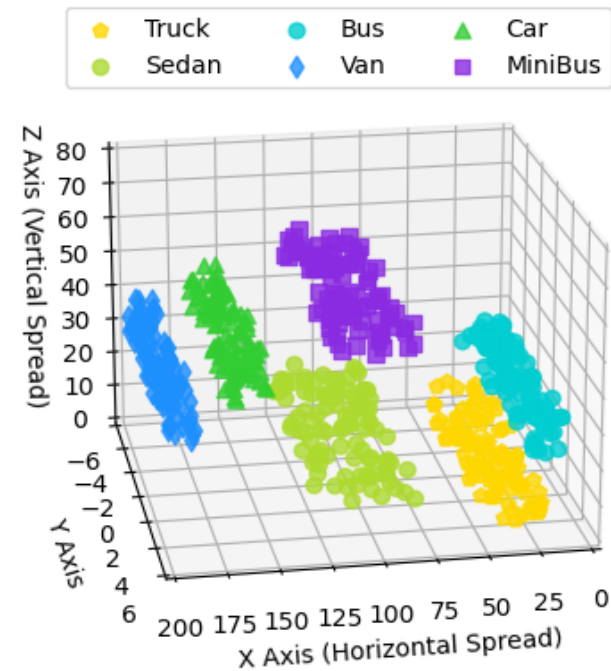

Supplement: Supplemental Information 3 [file peerj-cs-11-3270-s003.pdf]
